# LncRNA CASC 2 is upregulated in aphthous stomatitis and predicts the recurrence

**DOI:** 10.1186/s12903-019-0993-0

**Published:** 2020-01-14

**Authors:** Jiaping Lu, Naizheng Zhang, Chen Wu

**Affiliations:** 1 VIP Department, Shanghai Xuhui Dental Hospital, No.3, Lane 244, Pingjiang Road, Xuhui District, Shanghai, 200030 People’s Republic of China; 2 Shanghai Huangpu Dental Hospital, Shanghai, 200002 People’s Republic of China; 3 Department of Stomatology, Shanghai Baoshan Traditional Chinese Medicine-Integrated Hospital, Shanghai, 201900 People’s Republic of China

**Keywords:** Recurrent aphthous stomatitis, CASC2, IL-6, IL-18, Recurrence

## Abstract

**Background:**

Recurrent aphthous stomatitis (RAS) is a common oral disease with unknown molecular pathogenesis. Our preliminary microarray analysis revealed the altered expression of lncRNA Cancer Susceptibility Gene 2 (CASC2) in RAS. We therefore analyzed the role of CASC2 in RAS.

**Methods:**

In this study, plasma samples were obtained from RAS patients and healthy participants. Plasma levels of CASC2 were measured by RT-qPCR. Plasma levels of IL-6 and IL-18 were measured by enzyme-linked immunosorbent assay (ELISA). A follow-up study was performed to analyze the role of CASC2 in the recurrence of RAS.

**Results:**

In the present study, we found that lncRNA Cancer Susceptibility Gene 2 (CASC2), as well as pro-inflammatory factors interleukin 6 (IL-6) and interleukin 18 (IL-18), were upregulated in plasma of RAS patients compared with healthy participants. Plasma levels of lncRNA CASC2 were positively correlated with plasma levels of IL-6 and IL-18 in RAS patients but not in healthy participants. Compared with pre-treatment levels, plasma levels of lncRNA CASC2, IL-6 and IL-18 were reduced after recovery. A follow-up study showed that patients with high levels of lncRNA CASC2 had a significantly higher recurrence rate.

**Conclusion:**

LncRNA CASC 2 is upregulated in RAS and predicts the recurrence.

## Background

Recurrent aphthous stomatitis (RAS) is a commonly diagnosed and the recurrent inflammatory process in which ovoid or round painful ulcers of the oral mucosa recur [1, 2]. It has been well-established that food sensitivity, trauma, systemic conditions, nutritional deficiencies, immunological disorders and infections of Helicobacter pylori are closely correlated with the occurrence and progression of RAS [3]. However, the molecular pathogenesis of RAS remains unclear [4]. RAS patients can be treated by azathioprine systemic corticosteroids, pentoxifylline, systemic corticosteroids, or thalidomide [5]. However, more than 50% of patients with RAS will experience recurrence within 3 months after treatment and the long-term non-recurrence is rare.

It has been reported that the symptoms of RAS are also correlated with genetic alterations [6]. Long non-coding RNAs (lncRNAs, > 200 nt) have no ability in protein synthesis but participate in gene expression regulation at multiple levels [7, 8]. RAS is a type of inflammatory disease [9], in which lncRNAs have critical functions [10]. However, the functions of lncRNAs in RAS remain elusive. Cancer susceptibility gene2 (CASC2) is a well-characterized cancer-related lncRNA with oncogenic roles in many types of cancers including oral cancer [11–13]. Our preliminary microarray analysis revealed the altered expression of CASC2 in RAS. It has significant correlation with interleukin 6 (IL-6) and interleukin 18 (IL-18), as two critical inflammatory factors in RAS [14]. Therefore, this study was performed to analyze the involvement of CASC2 in RAS.

## Methods

### RAS patients and controls

This study passed the review board of the Shanghai Xuhui Dental Hospital Ethics Committee. Research subjects of the present study included 60 RAS patients (30 males and 30 females, 25 to 44 years’ old, 40.1±2.8 years’ old) and 60 healthy participants (30 males and 30 females, 25 to 44 years’ old, 40.3±2.7 years’ old) who were admitted to aforementioned hospital between May 2018 and May 2019. No other severe clinical disorders or other chronic diseases, such as diabetes, are diagnosed among the 60 RAS patients. Patients who were taking aspirin and any medications that may cause drowsiness were excluded. Pregnant females were also excluded. The 60 healthy participants received routine physiological exams at the aforementioned hospital. They were selected to match the age and gender distribution of the RAS group. All patients were informed of the principle of experimental design and they all signed informed consent.

### Therapies and follow-up

All patients were treated with thalidomide (100 mg/day) for 3 weeks. After 2 weeks of therapies, all patients recovered completely. It is known that thalidomide may cause life-threatening human birth defects, fatigue, sleepiness, weakness, confusion, and mood changes. To reduce side effects, aspirin, or products containing aspirin, other medications that may cause drowsiness and alcohol were avoided during medication. However, it is worth noting that thalidomide is not safe for everyone. Mild paralysis, paresthesia, and hypotension were observed in certain cases, while no severe adverse effects were observed. After that, the patients were followed up for 6 months in a monthly manner. The recurrence of RAS was recorded and used to plot a disease-free curve.

### Plasma preparation

On the day of admission (before the initiation of therapies), blood (5 ml) was extracted under fasting conditions from both patients and controls. The same amount of blood was also extracted from the patients after 3 weeks of treatment. All blood samples were centrifuged for 10 min at 1200 g and room temperature in ethylenediaminetetraacetic acid tubes to separate plasma. Plasma samples were immediately frozen in liquid nitrogen and stored in a liquid nitrogen sink before the following experiments.

### RNA extractions and RT-qPCR

Trizol reagent (Invitrogen) was used for all RNA extractions from cells and tissue samples from patients. To harvest miRNAs, 85% of ethanol was used to precipitate and wash RNA samples. LookOut® DNA Erase (Sigma-Aldrich) was used to digest all RNA samples to remove genomic DNAs. SuperScriptTM II reverse transcription kit (Invitrogen) was used to transcribe RNA samples into cDNAs, which were used as templates for performing qPCR assays using SYBR@ Premix Ex TaqTM (TaKaRa Bio Group). With GAPDH as endogenous control, the levels of CASC2 expression were measured. All PCR reactions were performed in triplicate and the 2^−ΔΔCT^ method was used to calculate the fold changes of gene expression.

### Enzyme-linked immunosorbent assay (ELISA)

Levels of IL-6 and IL-18 in plasma of both RAS patients and healthy participants were measured by ELISA using IL-6 Human ELISA Kit (Catalog # KHC0061, Invitrogen) and IL-18 Human ELISA Kit (Catalog # KHC0181, Invitrogen), respectively. Levels of IL-6 and IL-18 were expressed as pg/ml.

### Statistical analysis

SPSS 20.0 (IBM, SPSS, Chicago, IL, USA) was used for all data analysis, which was performed using mean values of data of 3 biological replicates. The unpaired t test was performed to analyze the differences between the two groups. Correlations were analyzed by linear regression. The paired t test was used to compare between two-time points within the same group. Kaplan-Meier method was used to plot disease-free curves of both high and low-level CASC2 groups (*n* = 30, cutoff value = the median level of CASC2 in the patient group after 3 weeks of treatment). Disease-free curves were compared by the log-rank test. *p* < 0.05 indicated a significant difference.

## Results

### CASC2, IL-6, and IL-18 were upregulated in RAS patients

**Fig. 1:**
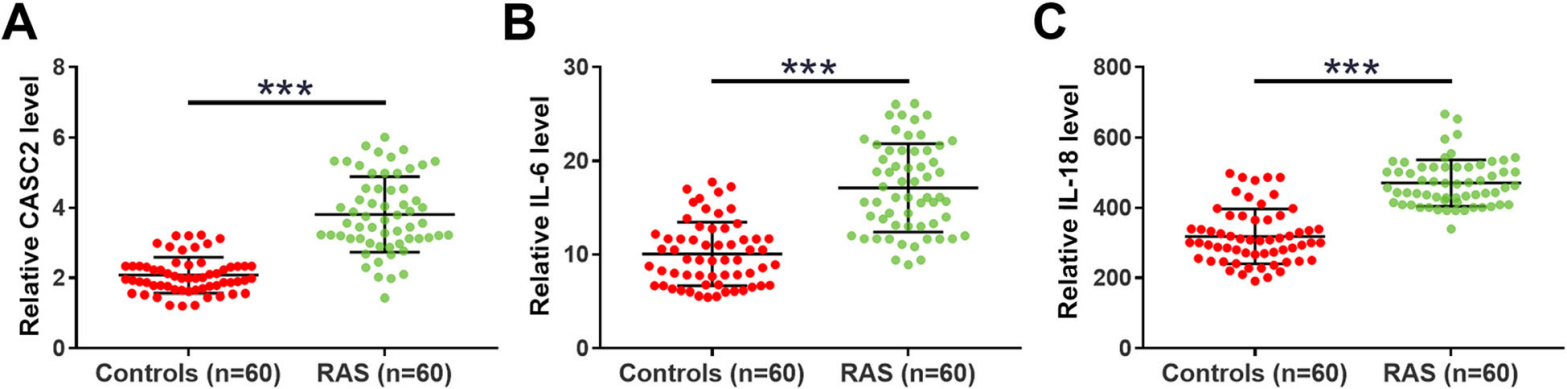
CASC2, IL-6 and IL-18 were upregulated in RAS patients. The differential expression of CASC2 (**a**), IL-6 (**b**), and IL-18 (**c**) was analyzed by performing qPCR and ELISA experiments. Data were compared between the two groups by performing an unpaired t-test. Three replicates were included in each measurement and data were expressed as mean values.***, *p* < 0.001

The differential expression of CASC2, IL-6, and IL-18 was analyzed by performing qPCR and ELISA experiments. Data were compared between the two groups by performing an unpaired t-test. It was observed that compared to the Control group, plasma levels of CASC2 (Fig. 1a), IL-6 (Fig. 1b) and IL-18 (Fig. 1c) were significantly higher in the RAS group (*p* < 0.001).

### CASC2 was positively correlated with IL-6 and IL-18 in RAS patients

**Fig. 2:**
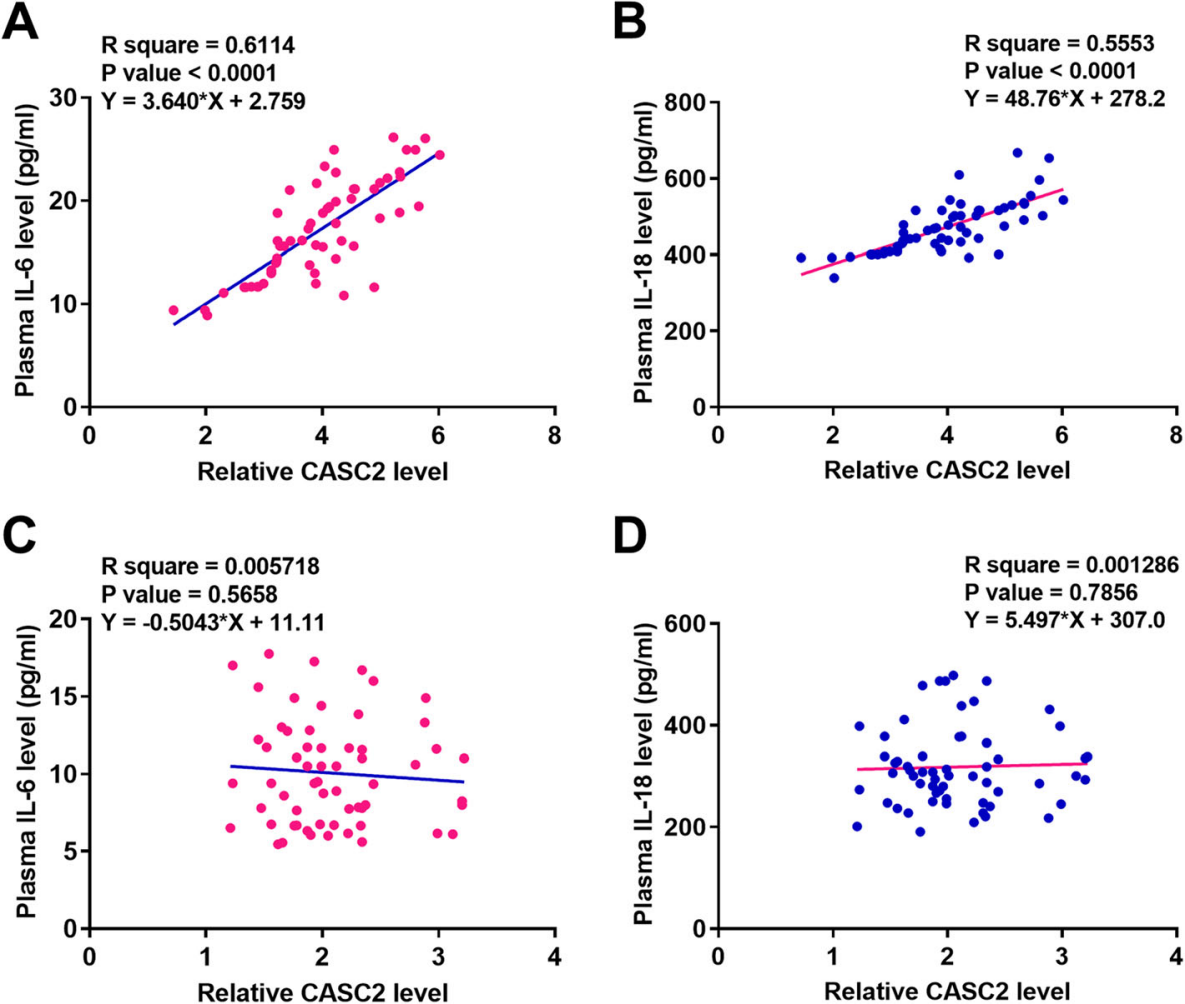
CASC2 was positively correlated with IL-6 and IL-18 in RAS patients. Correlations between CASC2 with IL-6 (**a**) and IL-18 (**b**) across RAS patients and the correlation between CASC2 with IL-6 (**a**) and IL-18 (**b**) across healthy participants were analyzed by linear regression

Correlations between CASC2 with IL-6 and IL-18 across RAS patients and healthy participants were analyzed by linear regression. It was observed that plasma levels of CASC2 were significantly and positively correlated with plasma levels of IL-6 (Fig. 2a) and IL-18 (Fig. 2b) across plasma samples from 60 RAS patients. In contrast, no significant correlations between plasma levels of CASC2 and plasma levels of IL-6 (Fig. 2c) and IL-18 (Fig. 2d) were found across plasma samples from 60 healthy participants.

### Plasma levels of CASC2, IL-6 and IL-18 were reduced after recovery

**Fig. 3:**
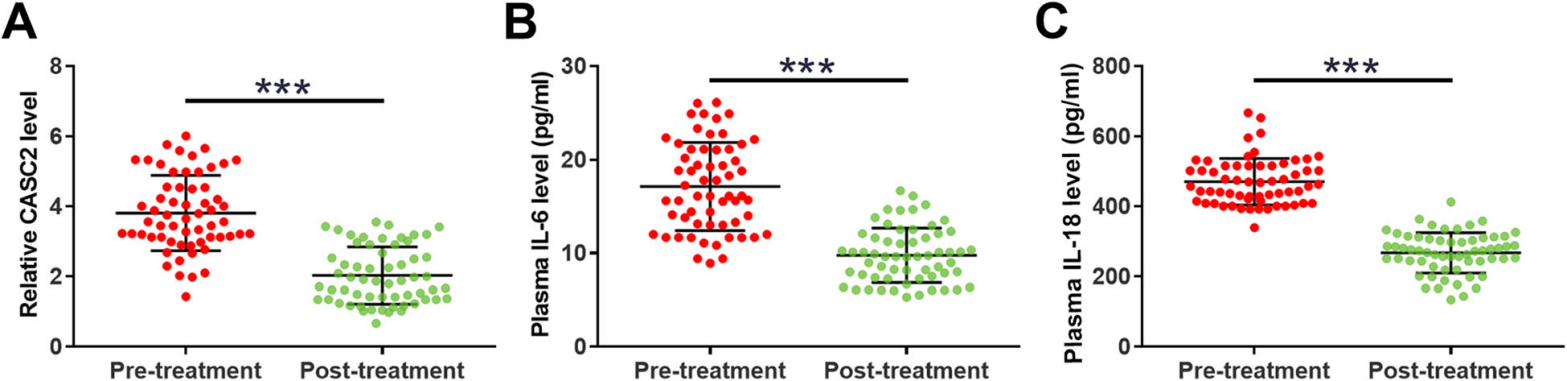
Plasma levels of CASC2, IL-6 and IL-18 were reduced after recovery. qPCR and ELISA were also performed to measure the plasma levels of CASC2 (**a**), IL-6 (**b**) and IL-18 (**c**) after 3 weeks of treatment. Comparisons between pre- and post-treatment levels were performed by performing a paired t-test. Each measurement was repeated 3 times and data for comparisons were the mean values of 3 biological replicates

qPCR and ELISA were also performed to measure the plasma levels of CASC2, IL-6, and IL-18 after 3 weeks of treatment. Comparisons between pre- and post-treatment levels were performed by performing a paired t-test. Comparing to pre-treatment levels, significantly reduced plasma levels of CASC2 (Fig. 3a), IL-6 (Fig. 3b) and IL-18 (Fig. 3c) were observed after 3 weeks of treatment (*p* < 0.001).

### High levels of CASC2 predicted the high recurrent rate of RAS

**Fig. 4:**
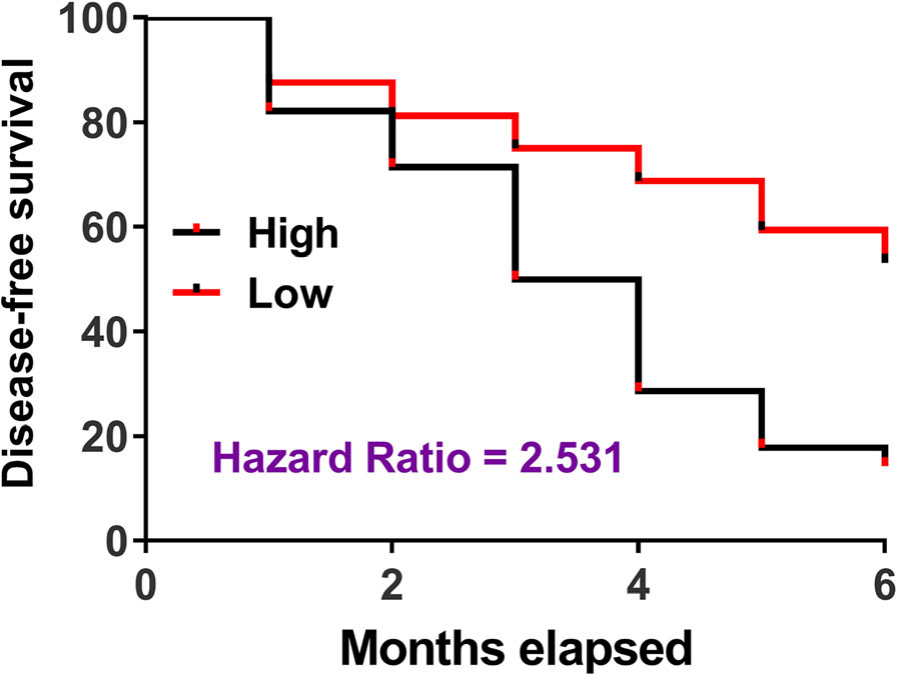
High levels of CASC2 predicted the high recurrent rate of RAS. The 60 patients were divided into high and low CASC2 levels groups (*n* = 30) with the median plasma level of CASC2 in RAS patients after 3 weeks of treatment as cutoff values. Kaplan-Meier method was used to plot disease-free curves of both high and low-level CASC2 groups Disease-free curves were compared by the log-rank test

The 60 patients were divided into high and low CASC2 levels groups (*n* = 30) with the median plasma level of CASC2 in RAS patients after 3 weeks of treatment as cutoff values. Disease-free curves were plotted and compared through the methods mentioned in the “statistical analysis” section. Comparing to patients in low CASC2 level group, patients in high CASC2 level group experienced a significantly low disease-free rate during 6 months’ follow-up (Fig. 4).

## Discussion

Although lncRNAs are important determinants in most, if not all human diseases, the functions of lncRNAs in RAS are unclear. This study explored the expression pattern of CASC2 in RAS and analyzed its prognostic values for this disease.

The functions and expression patterns of CASC2 have only been investigated in cancer biology, such as oral squamous cell carcinoma (OSCC) [11–13]. In OSCC CASC2 is upregulate and promotes cancer cell proliferation, invasion, and migration of cancer cells through the interactions with multiple pathways, such as programmed cell death protein 4 and cyclin-dependent kinase 1 [11–13]. In this study we first reported the upregulation of CASC2 in RAS, indicating the involvement of CASC2 in this disease.

Recurrence of RAS is common and in some cases, RAS can even recur multiple times within a year [15]. Therefore, it will be a great interest in the development of prognostic markers to predict the high-risk population. Some molecular markers, such as calprotectin, have been proven to be with predictive values for the recurrence of RAS [16]. However, the application of these prognostic markers is limited by low accuracy. In this study, we observed the downregulated CASC2 in RAS patients after treatment. In this study, all patients were treated with thalidomide (100 mg/day) for 3 weeks. We observed that, after 2 weeks of therapies, all patients recovered completely. Therefore, the course of treatment should be sufficient. In addition, a follow-up study revealed the association between high plasma levels of CASC2 in RAS patients after treatment and the high recurrent rate of RAS. Therefore, CASC2 may be used as a molecular marker to predict the treatment outcomes as well as the recurrence of RAS. However, the reliability of the prediction remains to be further tested by more clinical studies with a big sample size.

In this study observed the significant correlation between CASC2 with IL-6 and IL-18 only in RAS patients but not in healthy participants. Therefore, we speculate that some pathological mediators may mediate the interaction between CASC2 and IL-6 and IL-18. However, the mechanism of the potential interactions between them is still unclear. Future studies are needed to explore the possible mechanisms. In a recent study, Li et al. reported that CASC2 could inhibit autophagy [17]. Autophagy plays diverse pathological functions. Our future studies will explore the role of CASC2-regulated autophagy in RAS.

## Conclusion

CASC2 is upregulated in RAS and predicts the treatment outcomes and recurrence of RAS. The roles of CASC2 in RAS are likely related to inflammatory factors IL-6 and IL-18.

## Data Availability

The analyzed data sets generated during the study are available from the corresponding author on reasonable request.

## Abbreviations

lncRNAs: Long non-coding RNAs
RAS: Recurrent aphthous stomatitis
